# The high expression of TOP2A and MELK induces the occurrence of psoriasis

**DOI:** 10.18632/aging.205519

**Published:** 2024-02-20

**Authors:** Xinhua Zhu, Erjia Zhang, Li Qin

**Affiliations:** 1 Department of Dermatology, China Aerospace Science and Industry Corporation 731 Hospital, Beijing 100074, China

**Keywords:** TOP2A, MELK, psoriasis, differentially expressed genes

## Abstract

Background: Psoriasis is a chronic inflammatory skin disease. However, the influence of the TOP2A and MELK genes on psoriasis remains unclear.

Methods: Psoriasis datasets GSE166388 and GSE181318 were downloaded from the Gene Expression Omnibus (GEO) database generated from GPL570 and GPL22120. Differential gene expression (DEGs) was identified. Functional enrichment analysis, gene set enrichment analysis (GSEA), weighted gene co-expression network analysis (WGCNA), and immune infiltration analysis were conducted. The protein-protein interaction (PPI) network was constructed and analyzed. Gene expression heat map was generated. The most relevant diseases associated with core genes were determined through comparison with the Comparative Toxicogenomics Database (CTD) website. TargetScan was used to select miRNAs regulating central DEGs.

Results: A total of 773 DEGs were identified. According to Gene Ontology (GO) analysis, they were mainly enriched in mitochondrial gene expression, oxidative phosphorylation, mitochondrial envelope, mitochondria and ribosome. Kyoto Encyclopedia of Genes and Genomes (KEGG) analysis revealed that target cells were mainly enriched in metabolic pathways, proteasome, and oxidative phosphorylation. Seven core genes (TOP2A, NUF2, MELK, ASPM, DLGAP5, CCNA2, DEPDC1B) were obtained. The gene expression heatmap showed high expression of core genes (TOP2A, MELK) in psoriasis samples, while DEPDC1B, CCNA2, DLGAP5, NUF2, ASPM were lowly expressed in psoriasis samples. CTD analysis found that TOP2A and MELK were related to skin neoplasms, skin diseases, psoriasis, erythema, dermatitis, and infections.

Conclusion: TOP2A and MELK genes are highly expressed in psoriasis, and higher expression of TOP2A and MELK genes is associated with poorer prognosis.

## INTRODUCTION

Psoriasis is a chronic skin disorder that can occur on various parts of the body [1]. It is typically characterized by red patches on the skin covered with silver-white scales and may be accompanied by mild itching or discomfort [2]. The prevalence of psoriasis varies among different regions and populations. It tends to be higher in high-latitude areas such as Northern European countries, North America, and Australia, while lower rates are observed in Asia and Africa. However, generally, the prevalence falls within the range of 1% to 3%. It’s worth noting that there appears to be an increasing trend in psoriasis prevalence in recent years [3]. Psoriasis can affect individuals of any age group, but it most commonly first manifests during adolescence and early adulthood, with similar incidence rates in both males and females [4]. The pathological characteristics of psoriasis involve abnormal changes in skin and the immune system, with a key feature being the aberrant growth and differentiation of skin keratinocytes [5]. Psoriasis is associated with inflammatory reactions within the skin tissue, where T cells, macrophages, and other immune cells become overly active in affected areas, leading to inflammation and abnormal cell growth. It also exhibits abnormal blood vessel formation, which can increase blood flow to the affected area, further exacerbating inflammation and cell infiltration [6]. Psoriasis is considered an autoimmune disease, where the immune system erroneously attacks normal skin cells. While psoriasis typically does not pose a direct threat to life, it does carry some risks, as individuals with psoriasis may be more prone to cardiovascular diseases than the general population [7]. While there is currently no cure for psoriasis, there are various treatment approaches available to alleviate symptoms and manage the disease's progression. Treatment options include medication, phototherapy (the use of specific types of UV light to irradiate the skin), topical treatments, and lifestyle management [8]. The exact cause of psoriasis is not fully understood, but abnormal immune system activity, genetic factors, and environmental factors may all play a role in the development of the disease. Genetic factors play a crucial role in the pathogenesis of psoriasis, and individuals with psoriasis often have a family history of the condition [9, 10]. The genetic basis of psoriasis is primarily associated with variations in genes related to the human leukocyte antigen (HLA) loci [11]. The HLA system is one of the most important molecular complexes in the human immune system, participating in the recognition of self and foreign antigens by T lymphocytes [12]. Therefore, in-depth research into the molecular mechanisms of psoriasis is of great importance.

DNA Topoisomerase II Alpha (TOP2A) encodes an enzyme that participates in DNA topological changes, specifically the unwinding and winding of DNA strands, playing a crucial role in the structure and replication of DNA [13]. As skin cells are a continually renewing and regenerating cell type, the activity of TOP2A may play a significant role in the normal physiological processes of skin cells. Maternal Embryonic Leucine Zipper Kinase (MELK) is a kinase that plays a role in regulating biological processes such as the cell cycle, cell proliferation, and apoptosis [14]. In the skin, the balanced proliferation and differentiation of cells are crucial for maintaining the normal structure and function of the skin. Therefore, the aberrant expression of MELK may be associated with the pathogenesis of certain skin diseases.

Bioinformatics technology is an interdisciplinary field that combines biology, computer science, and data analysis to handle and analyze biological data [15]. The rapid advancement of bioinformatics technology has allowed researchers to better understand complex biological problems and has provided powerful tools for various fields, including medicine, drug development, ecology, and agriculture. With continuous technological advancements and interdisciplinary collaboration, bioinformatics will continue to play a crucial role in research, healthcare, and the biotechnology industry. Bioinformatics technology has wide-ranging applications, including accelerating life science research, improving medical practices, advancing drug development, and protecting ecosystems, offering numerous important advantages to the fields of science and health [16].

However, the relationship between the TOP2A and MELK genes and psoriasis is currently unclear. Therefore, this study aims to utilize bioinformatics technology to uncover the core genes associated with psoriasis compared to normal tissue. It will involve enrichment analysis and pathway analysis to understand the significant roles of the TOP2A and MELK genes in psoriasis using publicly available datasets.

## METHODS

### Psoriasis datasets

In this study, psoriasis datasets GSE166388 and GSE181318 profiles were downloaded from the Gene Expression Omnibus (GEO) database (http://www.ncbi.nlm.nih.gov/geo/) generated from GPL570 and GPL22120. GSE166388 [17] consists of 4 psoriasis samples and 4 normal tissue samples, GSE181318 comprises 3 psoriasis samples and 3 normal tissue samples. These datasets were utilized for the identification of differentially expressed genes (DEGs) associated with psoriasis.

### Batch effect removal

For the integration of multiple datasets and batch effect removal, we employed R software packages to process the datasets GSE166388 and GSE181318. Firstly, we merged the datasets using the R package inSilicoMerging (https://doi.org/10.1186/1471-2105-13-335), resulting in a merged matrix. Subsequently, we further utilized the R package limma (version 3.42.2) and its remove BatchEffect function to eliminate batch effects. This final step yielded a matrix devoid of batch effects, which was subsequently applied in subsequent analyses.

### Selection of differentially expressed genes (DEGs)

The R package “limma” was used for probe summarization and background correction of the merged matrix from GSE166388 and GSE181318. The Benjamini-Hochberg method was employed to adjust the raw *p*-values. The fold change (FC) was calculated using the false discovery rate (FDR). DEGs were filtered based on a cutoff of *p* < 0.05 and FC > 2. A volcano plot was generated for visualization.

### Functional enrichment analysis

Gene Ontology (GO, http://www.geneontology.org/) and Kyoto Encyclopedia of Genes and Genomes (KEGG, https://www.kegg.jp/) analyses are computational methods for assessing gene function and biological pathways. In this study, the selected list of differentially expressed genes was input into the KEGG API (https://www.kegg.jp/kegg/rest/keggapi.html) to obtain the latest gene annotations for KEGG Pathways, which served as the background. Genes were mapped to the background set, and enrichment analysis was conducted using the R package cluster Profiler (version 3.14.3) to obtain results of gene set enrichment. Additionally, gene GO annotations from the R package (version 3.1.0) were used, setting a minimum gene set size of 5 and a maximum gene set size of 5000. Statistical significance was defined as a *P* value of < 0.05 and an FDR of < 0.25.

Furthermore, the Metascape (https://metascape.org/) database provided comprehensive gene list annotation and analysis resources, with the option for visualized exports. We utilized the Metascape database (http://metascape.org/gp/index.html) for functional enrichment analysis of the aforementioned list of differentially expressed genes and for data export.

### GSEA

For Gene Set Enrichment Analysis (GSEA), we obtained the GSEA (https://www.gsea-msigdb.org/gsea/index.jsp) software (version 3.0) from the GSEA website (https://doi.org/10.1073/pnas.0506580102, http://software.broadinstitute.org/gsea/index.jsp). We divided the samples into two groups based on disease samples and normal samples and downloaded subsets from the Molecular Signatures Database (https://doi.org/10.1093/bioinformatics/btr260, http://www.gsea-msigdb.org/gsea/downloads.jsp) for evaluating relevant pathways and molecular mechanisms. This analysis was conducted based on gene expression profiles and phenotype grouping. The parameters used included a minimum gene set size of 5, a maximum gene set size of 5000, 1000 permutations, and statistical significance defined as a *P* value of < 0.05 and an FDR of < 0.25. Furthermore, we performed GO and KEGG analyses on the entire genome as per GSEA’s guidelines.

### Weighted gene co-expression network analysis (WGCNA)

Using the gene expression profile, the Median Absolute Deviation (MAD) of each gene was calculated, and the genes with the lowest MAD in the top 50% were excluded. The outlier genes and samples were removed by the R package WGCNA good Samples Genes method. Furthermore, WGCNA was used to construct a scale-free co-expression network. To classify genes with similar expression profiles into gene modules, the average linkage rank of the gene dendrograms was clustered according to TOM-based dissimilarity measures, with a minimum genome of 30. Sensitivity was set to: 3, for further analysis of modules, differences between module characteristic genes were calculated, a tangent line was selected as a module dendrogram, and some modules were merged. In addition, modules with a distance less than 0.25 were merged, and it is worth noting that grey modules were considered to be the set of genes that could not be assigned to any module.

### Construction and analysis of protein-protein interaction (PPI) networks

The Search Tool for the Retrieval of Interacting Genes (STRING) database (http://string-db.org/) is designed to collect, score, and integrate all publicly available protein-protein interaction (PPI) information sources, supplementing them with computational predictions. In this study, the list of differentially expressed genes was input into the STRING database to construct a predicted PPI network for core genes with a confidence score > 0.4. Cytoscape (http://www.cytoscape.org/) software is a tool that provides biological network analysis and two-dimensional visualization for researchers. It can be used to visualize and predict core genes from the PPI network generated from the STRING database. Import the PPI network into Cytoscape software, use MCODE to find the most relevant modules, and then calculate the best-related genes using five algorithms (MCC, MNC, DMNC, EPC, Degree) separately. Finally, visualize and export the list of core genes after taking the intersection.

### Gene expression heatmap

Using the R package heatmap, create a heatmap of the expression levels of core genes identified by five algorithms in the PPI network for GSE166388 and GSE181318. Visualize the expression differences of core genes between psoriasis and normal tissue samples.

### Immunoinfiltration analysis

CIBERSORT (http://CIBERSORT.stanford.edu/) is a commonly used method for calculating immune cell infiltration. The LM22 gene file is used to define 22 immune cell subtypes. We applied an integrated bioinformatics approach and used the CIBERSORT package to analyze batch-corrected matrices of GSE166388 and GSE181318. We utilized the principles of linear support vector regression to deconvolve the expression matrix of immune cell subtypes to estimate immune cell abundance. We selected samples with sufficient confidence, using a cutoff of *P* < 0.05.

### CTD analysis

The Comparative Toxicogenomics Database (CTD, http://ctdbase.org/) integrates a vast amount of data on interactions between chemicals, genes, functional phenotypes, and diseases. It provides significant convenience for research on disease-related environmental exposure factors and potential drug mechanisms. We input the core genes into the CTD website to identify the most relevant diseases associated with these core genes. Subsequently, we created radar plots of the expression differences for each gene using Excel.

### miRNA

TargetScan (http://www.targetscan.org) is an online database used for predicting and analyzing miRNA and their target genes. In the study, TargetScan was employed to screen for miRNAs regulating the central DEGs.

## RESULTS

### Differential gene expression analysis

**Figure 1:**
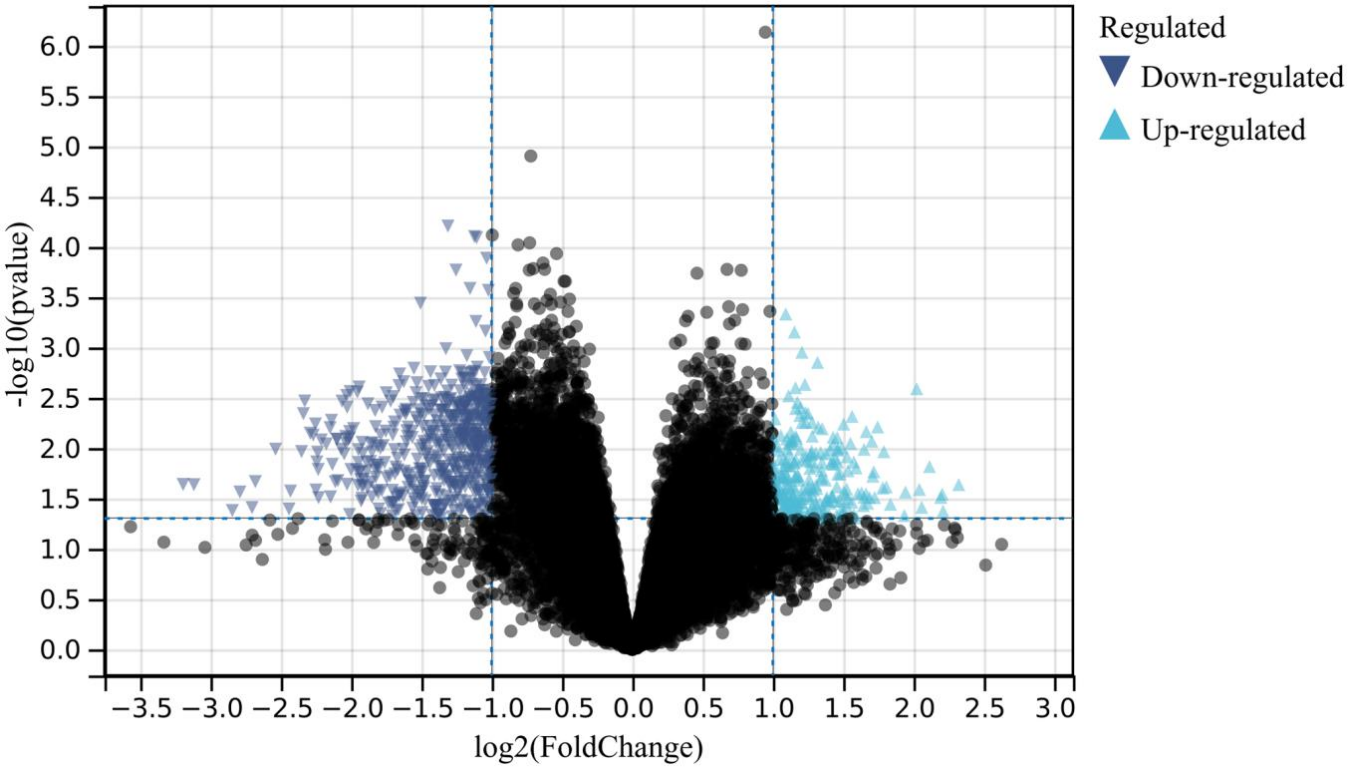
**Differential gene expression analysis.** A total of 773 differentially expressed genes (DEGs) were identified.

In this study, using predefined cutoff values, we identified a total of 773 differentially expressed genes (DEGs) from the batch-corrected merged matrices of GSE166388 and GSE181318 (Figure 1).

### Functional enrichment analysis

#### DEGs

**Figure 2:**
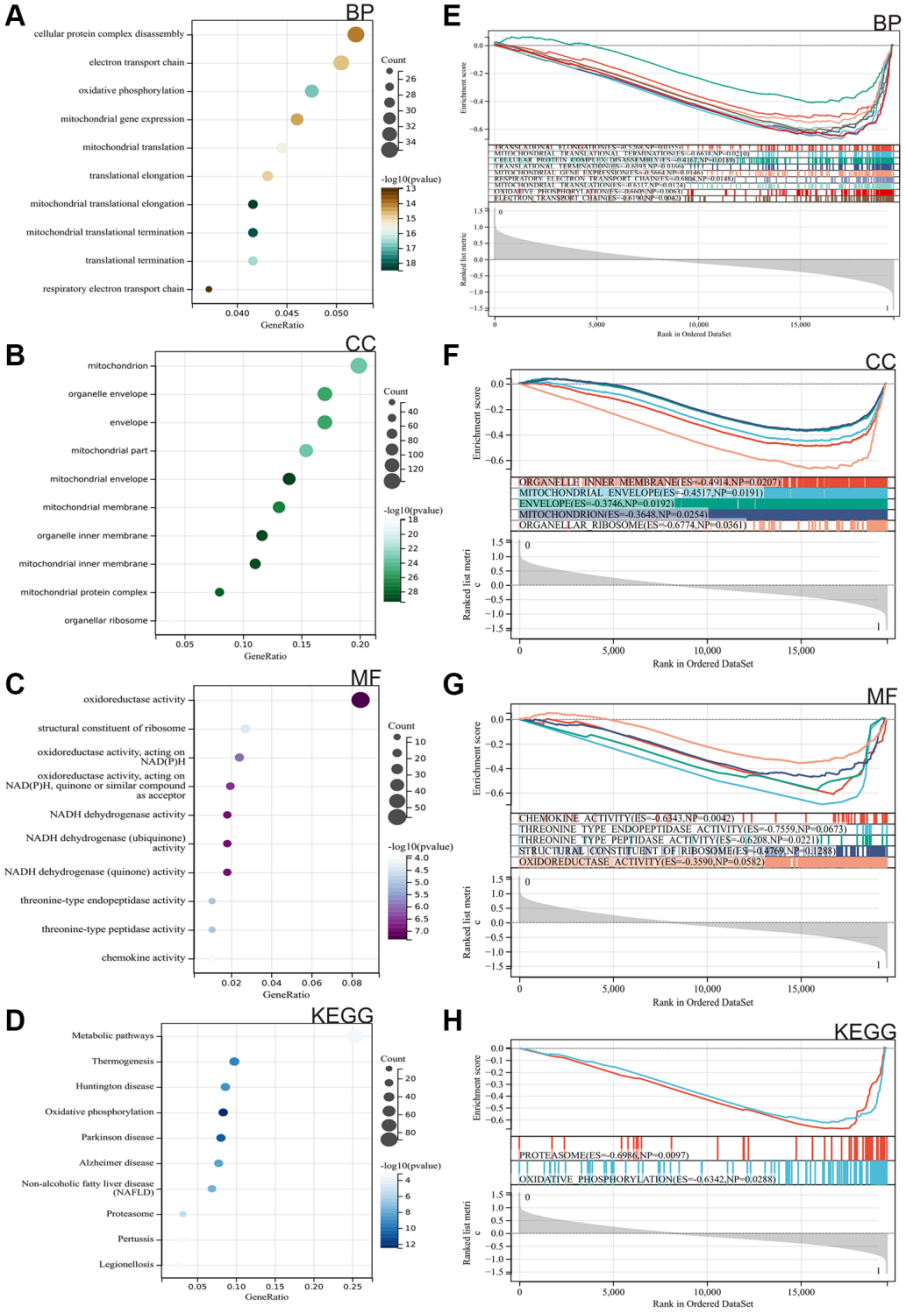
**Functional enrichment analysis.** (**A**–**C**) Gene Ontology (GO). (**D**) Kyoto Encyclopedia of Genes and Genomes (KEGG). (**E**–**H**) Gene Set Enrichment Analysis (GSEA).

We performed GO and KEGG analyses on these DEGs. According to the GO analysis, these genes were primarily enriched in translational elongation, mitochondrial translational termination, cellular protein complex disassembly, translational termination, mitochondrial gene expression, respiratory electron transport chain, mitochondrial translation, oxidative phosphorylation, electron transport chain, organelle inner membrane, mitochondrial envelope, envelope, mitochondria, organellar ribosome, chemokine activity, threonine-type endopeptidase activity, threonine-type peptidase activity, structural constituent of ribosome, oxidoreductse activity (Figure 2A–2C). KEGG analysis results indicated that target cells were mainly enriched in Metabolic pathways, Thermogenesis, Huntington disease, Oxidative phosphorylation, Parkinson disease, Alzheimer disease, Non-alcoholic fatty liver disease (NAFLD), Proteasome, Pertussis, Legionellosis (Figure 2D).

#### GSEA

Furthermore, we conducted GSEA enrichment analysis on the entire genome to identify potential enrichment items within non-differentially expressed genes and validate the results of differentially expressed genes. The intersection of enrichment items with the GO and KEGG enrichment items of differentially expressed genes is shown in the figure, primarily enriched in translational elongation, mitochondrial translational termination, cellular protein complex disassembly, translational termination, mitochondrial gene expression, respiratory electron transport chain, mitochondrial translation, oxidative phosphorylation, electron transport chain, organelle inner membrane, mitochondrial envelope, envelope, mitochondria, organellar ribosome, chemokine activity, threonine-type endopeptidase activity, threonine-type peptidase activity, structural constituent of ribosome, oxidoreductse activity, Oxidative phosphorylation and proteasome (Figure 2E–2H).

#### Metascape enrichment analysis

**Figure 3:**
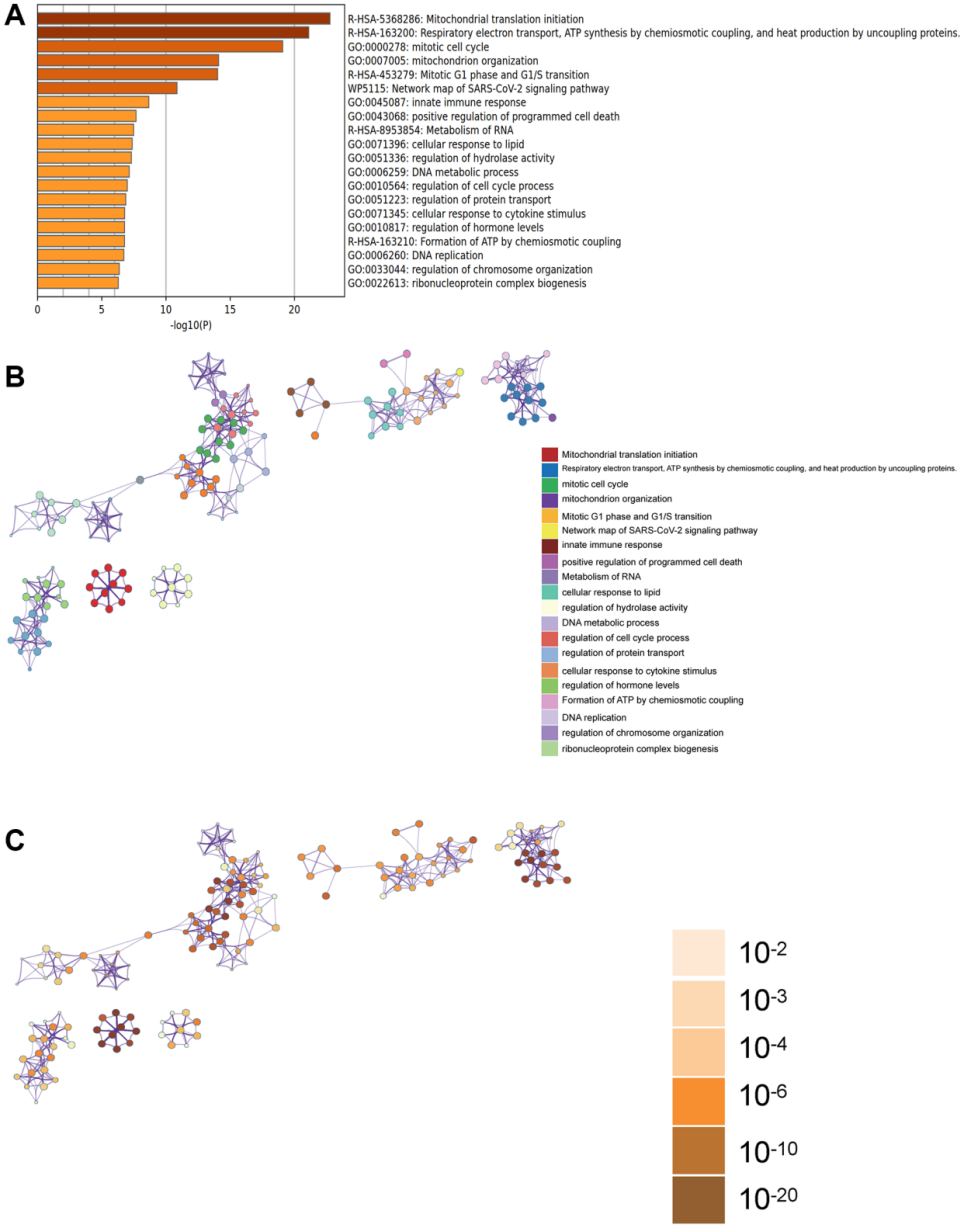
**Metascape enrichment analysis.** (**A**) Enrichment items in GO include mitochondrial organization, innate immune response, DNA metabolic process. (**B**, **C**) Enrichment networks colored by enrichment items and *p*-values.

In the Metascape enrichment analysis, GO enrichment items included mitochondrial organization, innate immune response, and DNA metabolic processes (Figure 3A). We also generated enrichment networks colored by enrichment items and *p*-values to visualize associations and confidence levels (Figure 3B, 3C).

### WGCNA

**Figure 4:**
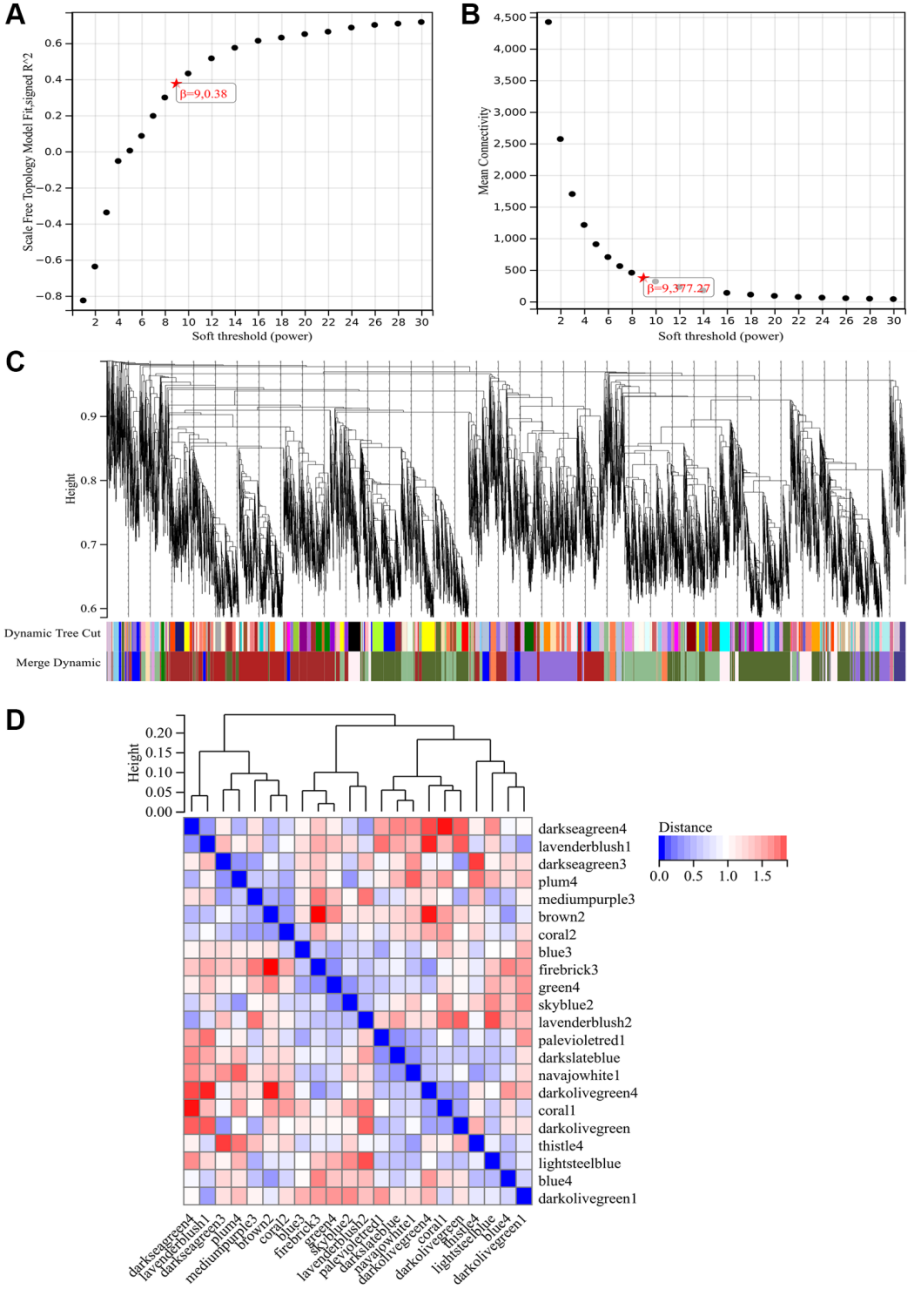
**WGCNA.** (**A**) β = 9, 0.38. (**B**) β = 9, 377.27. (**C**) Construction of hierarchical clustering tree for all genes and generation of important modules. (**D**) Analysis of interactions between modules.

**Figure 5:**
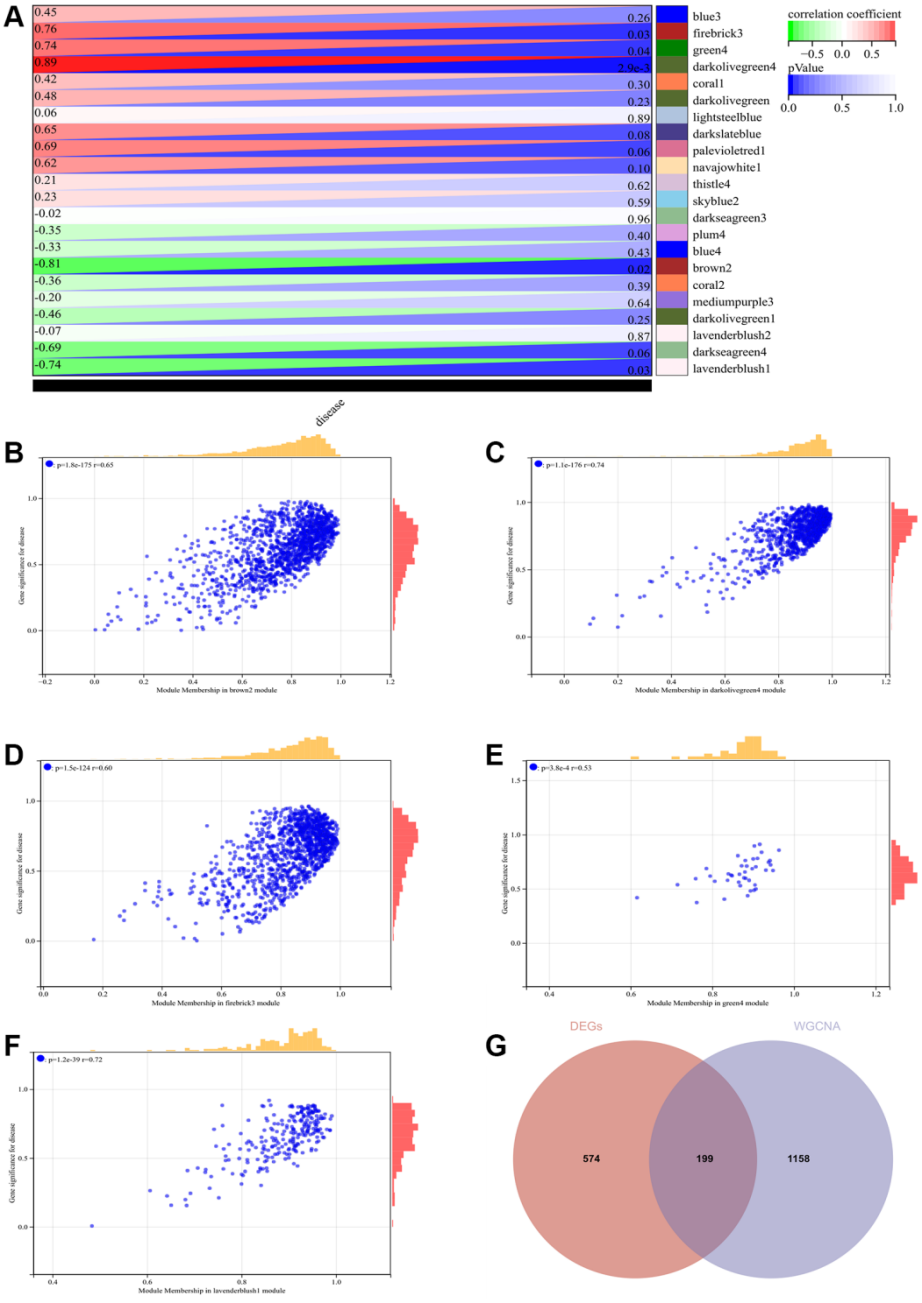
**WGCNA.** (**A**) Heatmap of module-trait correlations. (**B**–**F**) Scatter plot of correlation between GS and module membership (MM) for relevant hub genes. (**G**) Venn diagram illustrating the intersection.

The selection of the soft threshold power is a critical step in WGCNA analysis. Network topology analysis was performed to determine the soft threshold power. In the WGCNA analysis, the soft threshold power was set to 9 (Figure 4A, 4B). A hierarchical clustering tree of all genes was constructed, and significant modules were generated. The interactions between these modules were analyzed (Figure 4C, 4D). A heatmap of module-trait correlations (Figure 5A) and scatterplots of the correlation between GS and module membership (MM) for related hub genes (Figure 5B–5F) were generated.

We calculated the expression correlation between module feature vectors and genes to obtain MM. Based on the cutoff criterion (|MM| > 0.9), 1358 highly connected genes were identified as hub genes within clinically significant modules.

We also generated a Venn diagram by intersecting the modules selected through WGCNA with DEGs, which was used for creating and analyzing the protein-protein interaction network (Figure 5G).

### Protein-protein interaction (PPI) network construction and analysis

**Figure 6:**
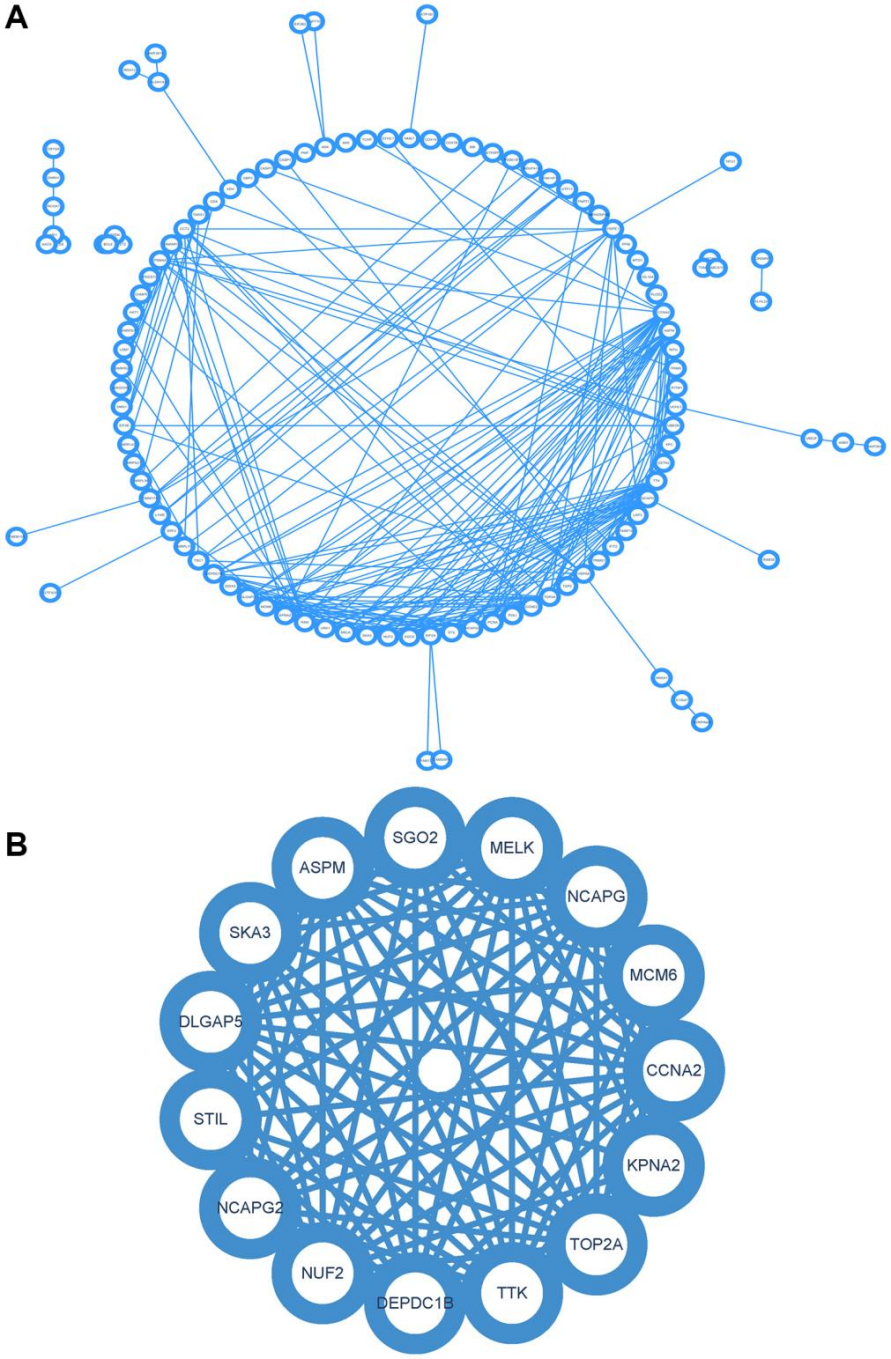
**Construction and analysis of protein-protein interaction (PPI) network.** (**A**) PPI network of DEGs. (**B**) Core gene clusters.

**Figure 7:**
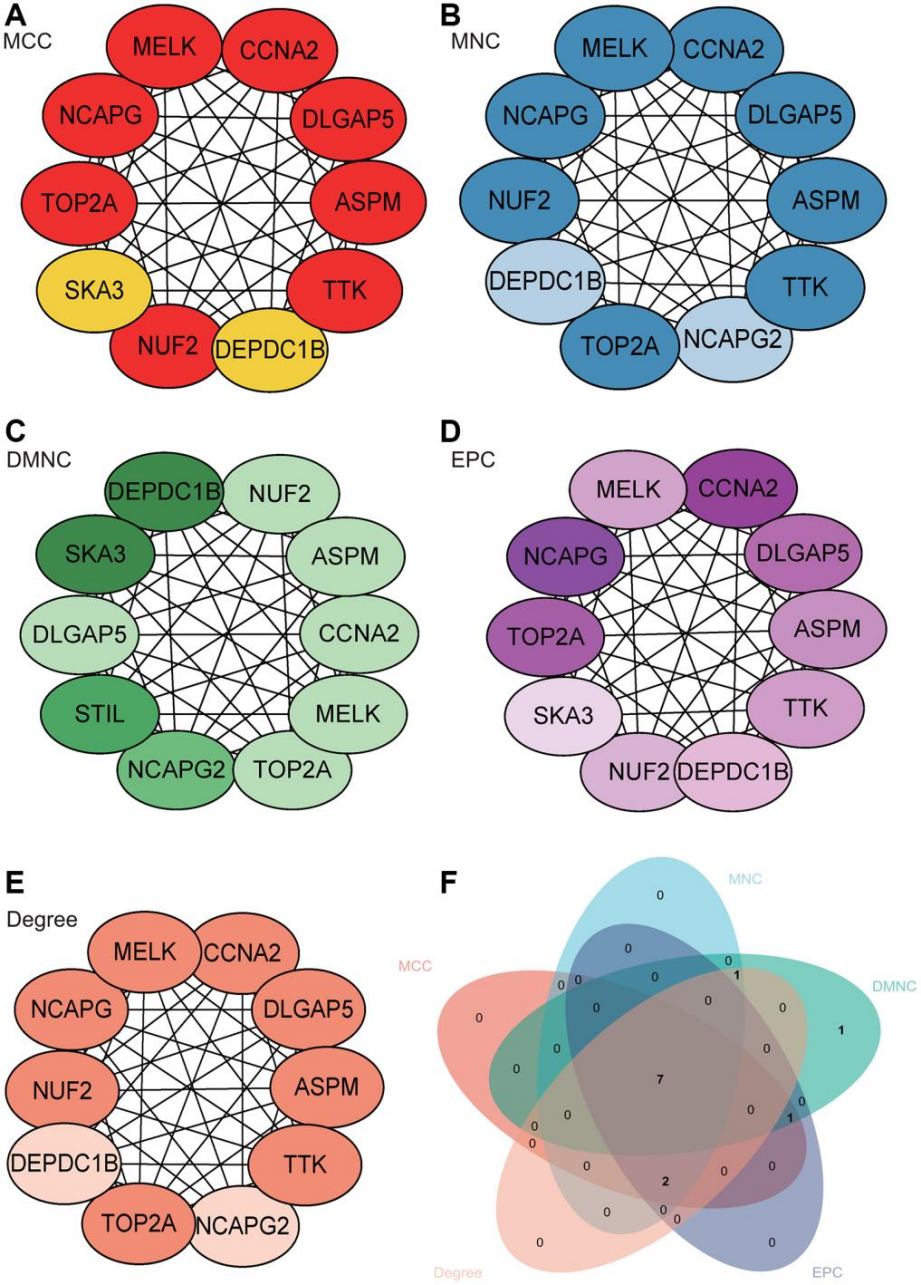
**Construction and analysis of protein-protein interaction (PPI) network.** (**A**–**E**) Identification of central genes using five different algorithms. (**F**) Venn diagram showing the intersection.

The PPI network of DEGs was constructed using the STRING online database and analyzed using Cytoscape software (Figure 6A). Core gene clusters were obtained (Figure 6B). Central genes were identified using five different algorithms (Figure 7A–7E), and the intersection was obtained using a Venn diagram (Figure 7F), resulting in 7 core genes (TOP2A, NUF2, MELK, ASPM, DLGAP5, CCNA2, DEPDC1B).

### Gene expression heatmap

**Figure 8:**
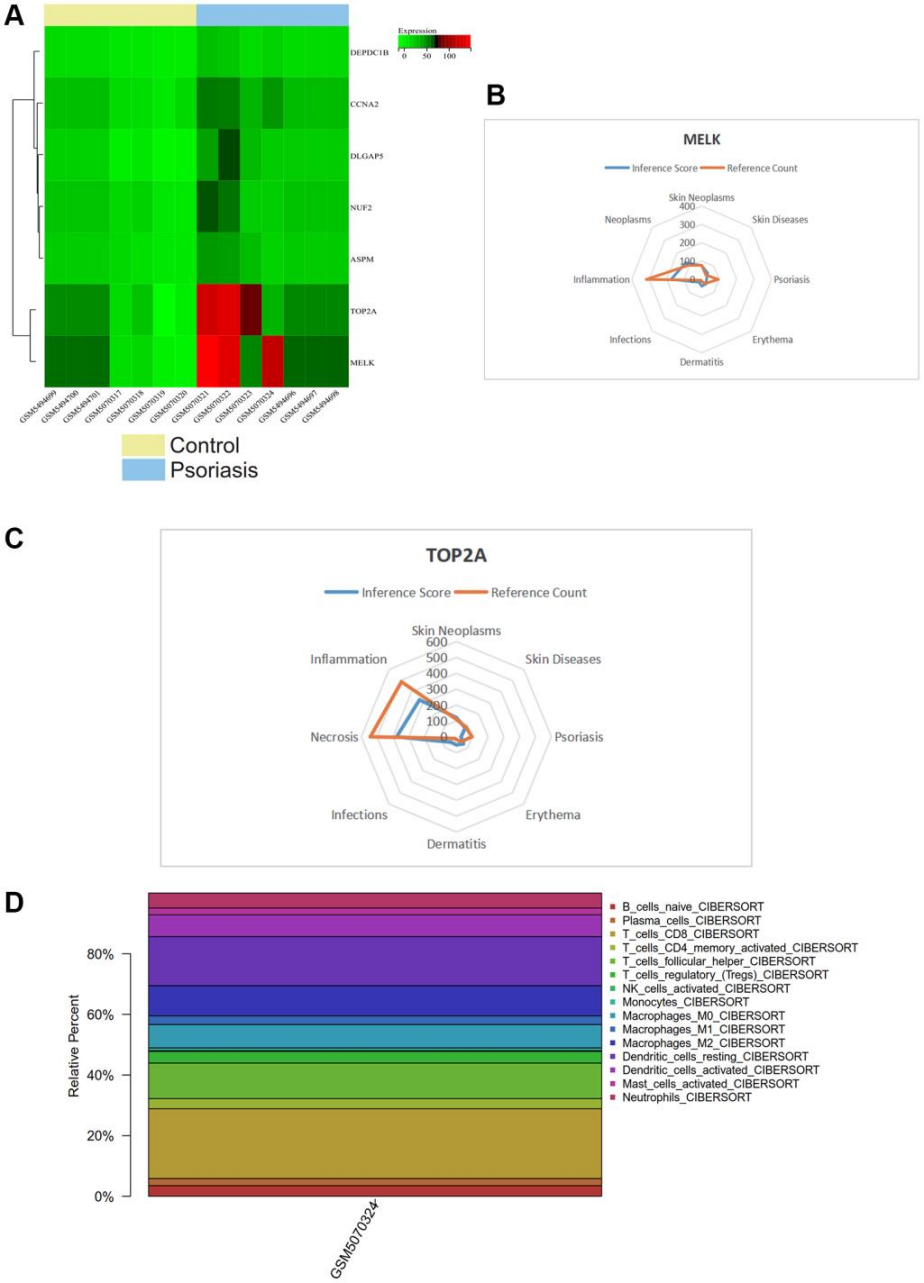
(**A**) Heatmap of gene expression levels. (**B**, **C**) CTD analysis related to TOP2A, MELK, and their association with skin tumors, skin diseases, psoriasis, erythema, dermatitis, and infection. (**D**) Immune infiltration analysis showing the proportion of immune cells in the whole gene expression matrix.

We visualized a heatmap of core gene expression in the samples (Figure 8A). We found that core genes (TOP2A, MELK) were highly expressed in psoriasis samples and lowly expressed in normal tissue samples, while DEPDC1B, CCNA2, DLGAP5, NUF2, ASPM were lowly expressed in psoriasis samples. Core genes (TOP2A, MELK) may play a regulatory role in psoriasis.

### CTD analysis

In this study, we input the core gene list into the CTD website to identify diseases related to core genes, improving our understanding of gene-disease associations. We found that TOP2A and MELK were associated with skin neoplasms, skin diseases, psoriasis, erythema, dermatitis, and infections (Figure 8B, 8C).

### Immune infiltration analysis

We used the CIBERSORT package to analyze the batch-corrected merged matrices of GSE166388 and GSE181318 and obtained the proportion of immune cells in the whole gene expression matrix at a 95% confidence level (Figure 8D). The results showed a higher proportion of T_cells_CD8.

### Prediction and functional annotation of miRNAs associated with Hub genes

In this study, we input the hub gene list into TargetScan to identify related miRNAs, enhancing our understanding of gene expression regulation (Table 1). We found that hsa-miR-144-3p was associated with the TOP2A gene, and hsa-miR-802 was associated with the MELK gene.

**Table 1:** A summary of miRNAs that regulate hub genes.

	**Gene**	**MIRNA**
**1**	**TOP2A**	hsa-miR-144-3p
**2**	**MELK**	hsa-miR-802

### Data availability

The datasets generated during and/or analyzed during the current study are available from the corresponding author on reasonable request.

## DISCUSSION

Psoriasis is a relatively common autoimmune skin disease that can lead to nutrient loss, joint and organ damage, and systemic inflammation, often triggered by infections [18]. Psoriasis also has a genetic component and is characterized by its high resistance to treatment, frequent relapses, and associations with conditions such as metabolic syndrome, psoriatic arthritis, and organ damage, including liver and kidney impairment, and even symptoms of heart failure in severe cases [19]. In-depth exploration of the molecular mechanisms of psoriasis is crucial for targeted drug development. The main findings of this study indicate that the TOP2A and MELK genes are highly expressed in psoriasis, and higher expression of TOP2A and MELK is associated with a worse prognosis.

TOP2A represents the DNA Topoisomerase IIα gene, which encodes a protein belonging to the DNA topoisomerase family [20]. DNA topoisomerases (TOP) are essential nuclear enzymes that primarily change the topology of DNA through catalysis. DNA topoisomerases play critical roles in managing DNA structure and topological configurations within cells, including untangling DNA helices and relieving tension in supercoiled DNA. They are involved in processes such as chromosome condensation, separation of chromatin monomers, and the release of torsional stress during DNA transcription and replication [21].

TOP2A is a gene that encodes DNA Topoisomerase IIα, a protein crucial for DNA topology changes during cell division, DNA replication, and maintenance of DNA structure. It mediates the cleavage and reconnection of DNA strands, allowing for the proper replication, separation, and distribution of chromosomes to two new cells, which are essential steps in cell division and growth [22]. TOP2A functions by mediating DNA double-strand cleavage (breakage and reconnection), promoting the formation of enzyme-DNA cleavage complexes, shifting the balance toward the stabilization of these complexes, extending their half-life and making them more stable, interfering with TOP2A-mediated DNA reconnection reactions, causing DNA single or double-strand breaks, affecting DNA replication, and triggering apoptosis, leading to cell death [23]. Abnormal expression or mutations in the TOP2A gene may result in DNA damage and repair issues. The study have suggested that TOP2A may play a crucial role in the development of melanoma [24].

Psoriasis is an autoimmune skin disease with a complex interplay of various genetic, immunological, and environmental factors contributing to its pathogenesis. The immune system plays a significant role in the development of psoriasis [25]. Meanwhile, the protein encoded by the TOP2A gene is involved in DNA repair and cell division. While the TOP2A gene may not have a direct association with psoriasis, it could potentially play a role in the pathogenesis of psoriasis through pathways related to the immune system and cell cycle regulation.

Maternal Embryonic Leucine Zipper Kinase (MELK) is a cell cycle-dependent, evolutionarily conserved serine/threonine protein kinase, belonging to the AMPK family, located on chromosome 9p13.2. It is widely expressed in various cell types, including embryonic tissues and various adult tissues [26]. MELK is involved in multiple cellular processes, including cell cycle regulation, cell proliferation, cell differentiation, cell survival, and also plays roles in splicing complex assembly, gene expression, embryonic development, and hematopoiesis [27]. MELK is closely associated with cell cycle regulation, proliferation, mitosis, and splicing complex assembly [28]. MELK mRNA levels increase during mitosis [29]. MELK plays a crucial role in cell division, cell proliferation, and cell cycle regulation in organ-specific stem cells from non-mammalian species and is crucial for organogenesis, stem cell proliferation, and cell cycle regulation in mammalian systems [30].

Studies have indicated that MELK is associated with metastasis in triple-negative breast cancer [31]. Inhibition of MELK has shown potent anti-leukemic effects in chronic lymphocytic leukemia, making it a candidate therapeutic target [32]. MELK is explored as a potential therapeutic target for cancer [33]. MELK has been identified as an adverse prognostic marker and therapeutic target in osteosarcoma [34]. MELK is a potential therapeutic target and biomarker for lung adenocarcinoma [35]. MELK is often highly expressed in melanoma patients' samples compared to normal skin tissue. Dual targeting of AMPK/MELK is used for the treatment of novel melanoma [36]. MELK has close connections to immune checkpoints, and targeting MELK can enhance the effectiveness of immunotherapy [37]. Therefore, it is speculated that the MELK gene may play an important role in the development of psoriasis.

This study holds potential clinical significance, as the expression levels of TOP2A and MELK may serve as potential biomarkers for assessing the condition and prognosis of psoriasis. Elevated expression levels may be associated with disease progression and severity. Precise personalized treatment strategies could consider the gene expression information of TOP2A and MELK to better tailor therapeutic approaches. The research findings are statistically significant and hold importance in both biological and clinical contexts. These results can be extrapolated to a broader population or clinical practice, contributing to the understanding of the disease's pathogenesis or principles of treatment effectiveness. The research outcomes have practical implications for the medical management, treatment decisions, or prognosis assessment of patients. For both patients and healthcare professionals, understanding the background and methodology of the study, along with the clinical significance of the research findings, is crucial for making informed medical decisions.

Although this study conducted rigorous bioinformatics analysis, there are still some limitations. Animal experiments involving gene overexpression or knockout were not performed to further validate the functions of the identified genes. Therefore, future research should explore this aspect in more detail.

## CONCLUSION

The expression levels of TOP2A and MELK genes are associated with psoriasis. TOP2A and MELK genes are highly expressed in psoriasis, and higher expression of TOP2A and MELK is associated with a worse prognosis. TOP2A and AURKA genes also play important roles in psoriasis and may serve as molecular targets for precision therapy, providing new directions for further research into their mechanisms.
